# SOCIAL ISOLATION AND OLDER ADULTS: WHAT ROLE CAN TECHNOLOGY PLAY?

**DOI:** 10.1093/geroni/igz038.770

**Published:** 2019-11-08

**Authors:** Sara J Czaja

**Affiliations:** Weill Cornell Medicine, New York, New York, United States

## Abstract

Social isolation and loneliness are prevalent among older adults especially those who live in rural locations, have mobility restrictions, are in the older cohorts, live alone or live in residential institutions such as assisted living facilities or nursing homes. The detrimental consequences of isolation and loneliness on physical, cognitive, and emotional health are well documented. Technology applications such as the email, social media sites and online support groups hold promise in terms of enhancing engagement and providing support to older people and mitigating the negative impact of isolation and enhancing quality of life. Recent data indicate that use of these types of applications is increasing among older adults but there is still an age-related digital decline. This presentation will present findings from CREATE and other trials regarding the access to and use of these applications among older adults and the resultant impact on the social connectivity, loneliness and social support.

